# Elevated urinary glycosaminoglycans in Staphylococcus aureus bacteraemia with endovascular source

**DOI:** 10.1099/jmm.0.002169

**Published:** 2026-05-21

**Authors:** Dan F. Smelter, Cecilia F. Volk, Ravi Gandhi, George Sakoulas, Warren E. Rose

**Affiliations:** 1 College of Pharmacy, University of Texas at Austin, Austin, TX, USA; 2 Department of Medicine, UT San Antonio, San Antonio, TX, USA; 3 School of Pharmacy, University of Wisconsin-Madison, Madison, WI, USA; 4 Department of Pharmacy, UW Health, Madison, WI, USA; 5 Sharp Rees Stealy Medical Group, San Diego, CA, USA; 6 School of Medicine, University of California San Diego, La Jolla, CA, USA

**Keywords:** bacteraemia, endocarditis, endovascular, glycocalyx, glycosaminoglycan, Methicillin-susceptible Staphylococcus aureus

## Abstract

The differentiation of endovascular sources of *Staphylococcus aureus* bacteraemia from non-endovascular infection carries significant prognostic information that can be used to therapeutically stratify patients. The endovascular glycocalyx is a protective barrier composed of glycosaminoglycans (GAGs) that can be degraded and excreted in the urine from endothelial injury occurring in endovascular infections, but not in non-endovascular infections. To determine whether patients who had *S. aureus* endovascular infections, including endocarditis, had increased urinary glycocalyx shedding suggestive of endothelial damage, urine samples from 55 patients with bacteraemia caused by *S. aureus* were assessed for GAG content. Patients with endovascular source bacteraemia were compared to those from non-vascular source bacteraemia (e.g. prosthetic joint infections, pyomyositis and cellulitis) for GAG content. As expected, patients with *S. aureus* bacteraemia stemming from endovascular foci of infection showed increased GAG content in the urine (24.11±4.9 g GAG/mol creatinine, *n*=22) compared to those with non-vascular infections (13.65±1.6 g GAG/mol creatinine, *n*=33, *P*=0.024). Further analysis of GAG composition in urine revealed differential presence of GAGs between these two groups. In the first pilot study of its kind, we found that the measurement of GAG in the urine of patients with *S. aureus* bacteraemia shows promise for clinical risk stratification to identify high-risk endovascular infections in order to guide clinical diagnostic and therapeutic decision-making. Larger studies will be needed to determine relevant quantitative cut-off values.

## Data Summary

The datasets used and/or analysed during the current study are available from the corresponding author on reasonable request.

## Introduction

The endovascular glycocalyx is a functional bulwark composed of polysaccharides and proteins on the luminal surface of vascular endothelial cells. In addition to being a physical barrier between the vessel lumen and endothelial cell membrane, the glycocalyx modulates vascular homeostasis through regulation of vascular permeability, mechanosensing and leucocyte transmigration [1,2]. The proteoglycans and glycoproteins of the glycocalyx are dynamically regulated through constant variations in degradation and biosynthesis rates, and shedding of glycosaminoglycans (GAGs) from the endovascular glycocalyx results in excretion of GAGs into the urine of both healthy and sick individuals [3].

While GAGs in the urine may originate from the renal tubules and glomeruli in patients with renal disease [4], other specific disease states show increased vascular damage and destruction of the glycocalyx, resulting in increased GAG shedding into the urine [5]. This includes both pathological states and pathogens that can induce destruction of the endovascular layer, leading to GAG release and excretion in the urine of patients. For example, glycocalyx breakdown by matrix metalloproteinases, heparanases and hyaluronidases thins the protective layer, increases permeability and enhances tissue oedema in sepsis [6]. Furthermore, in patients with *Plasmodium falciparum* malaria, both disease severity and mortality correlate with increased urine GAG concentration [7]. In children with cerebral malaria, increased urine GAG content is associated with elevated plasma concentrations of cytokines such as TNF-*α* and IL-10, suggesting that disease severity and vascular damage may be mirrored by urine GAG levels [8]. Furthermore, overall GAG content in the urine correlates with GAG burden found in the serum of patients in the setting of many different disease states [9,10]; however, the individual content of the different GAGs may vary [9,11]. Collectively, these findings suggest that vascular endothelial damage as a process of disease pathogenesis may result in the accumulation of GAGs into the urine. The feasibility of obtaining urine in patients as a potentially low-cost, non-invasive early marker for disease severity provides a rational option over the more invasive, analytically complex measurement from the blood matrix.

*Staphylococcus aureus* is prominently responsible for a wide spectrum of diseases, including primary endovascular infections such as infective endocarditis (IE). Once in the bloodstream, *S. aureus* can subvert the host immune system through endothelial cell adhesion and invasion, surviving intracellularly where it can lead to persistent bacteraemia or relapse [12]. *S. aureus* adhesion proteins (collectively referred to as microbial surface components recognizing adhesive matrix molecules), such as those that bind to host fibronectin and fibrinogen, are expressed in greater quantities among *S. aureus* from persistent bacteraemia compared to rapidly resolving bacteraemia [13]. *S. aureus* persistent bacteraemia is associated with increased mortality and poorer clinical outcomes, and early definitive therapy improves patient survival [14].

Unfortunately, current treatment standards fail to address the early recognition of complex *S. aureus* bacteraemia, with the persistent cases declaring themselves simply by treatment failure before antibiotic therapy escalation. Such delays in recognition of complex endovascular infections place patients at increased risk of poor outcomes, including death. Indeed, mortality from *S. aureus* bacteraemia remains unchanged for decades due to the ‘one size fits all’ approach without attempts to differentiate high-risk patients at the time of initiation of antibiotics. Therefore, early biomarkers of high-risk endovascular *S. aureus* infection are desperately needed. We hypothesized that GAG content in the urine may be a diagnostic indicator of glycocalyx damage in *S. aureus* bacteraemia from an endovascular source, allowing earlier detection.

## Methods

This study was part of an ongoing biorepository of patients with *S. aureus* bacteraemia between the ages of 18 and 89 presenting at UW Health in Madison, WI. Using fresh, prospectively collected urine samples from 55 patients presenting with *S. aureus* bacteraemia from February 2020 to March 2022, as well as urine from ten healthy controls between the ages of 20 and 50 years, we first quantified the urinary creatinine by a colorimetric detection kit (Invitrogen™, #EIACUN). After normalization of the urine samples based on creatinine concentration, we then quantified urine GAG via colorimetric assay. The cationic dye, 1,9-dimethyl-methylene blue (DMMB), specifically binds to sulphated GAGs, and urine GAG was quantified by measuring absorption at 525 nm as previously described (Sigma-Aldrich, #341088) [15]. Briefly, 200 µl of the DMMB reagent (containing 1.6 mg DMMB, 305 mg glycine, 160 mg NaCl, 54 µl glacial acetic acid and bring to 100 ml with deionized water) was added to 8 µl of the sample in a 96-well plate, and the absorption was read immediately [11,16, 17]. Using patient electronic medical records, we then compared the urine GAG content from bacteraemia with an endovascular source (including IE, with and without reported intravenous drug usage) to those from non-vascular sources (e.g. prosthetic joint infections, pyomyositis and cellulitis) along with GAG content by patient stratifications of age, mortality and bacteraemia duration.

To visualize the sulphated GAG species present in the urine samples from patients with *S. aureus* bacteraemia, 12 samples (6 from patients with IE and 6 with bacteraemia from a non-vascular source) were randomly selected for isolation and separation via gel electrophoresis as described previously, with slight modifications [18]. In brief, urine samples were desalted and concentrated using Amicon ULTRA 3 kDa filters (#UFC5003), prepared and ran using polyacrylamide gel electrophoresis on a 4–15% gel (BIO-RAD 4–15% Criterion TGX Precast Midi Gel, #5671084). The sulphated GAGs were stained using 0.5% Alcian Blue 8 GX in 2% acetic acid and then washed and destained with deionized water prior to visualization on an Edvotek white light box. To verify proper staining of sulphated targets, dermatan sulphate and oversulphated chondroitin sulphate (European Pharmacopoeia Reference Standard, #Y0001321) was used as a positive control with the negative control being buffer plus loading dye.

Statistical analysis was performed using GraphPad Prism (GraphPad Software, San Diego, CA). Fisher’s exact test was used for categorical variables and Student’s t-test or Mann–Whitney U test for parametric or nonparametric continuous data, respectively, with a two-sided *P* value of ≤0.05 for statistical significance.

## Results

**Fig. 1.**
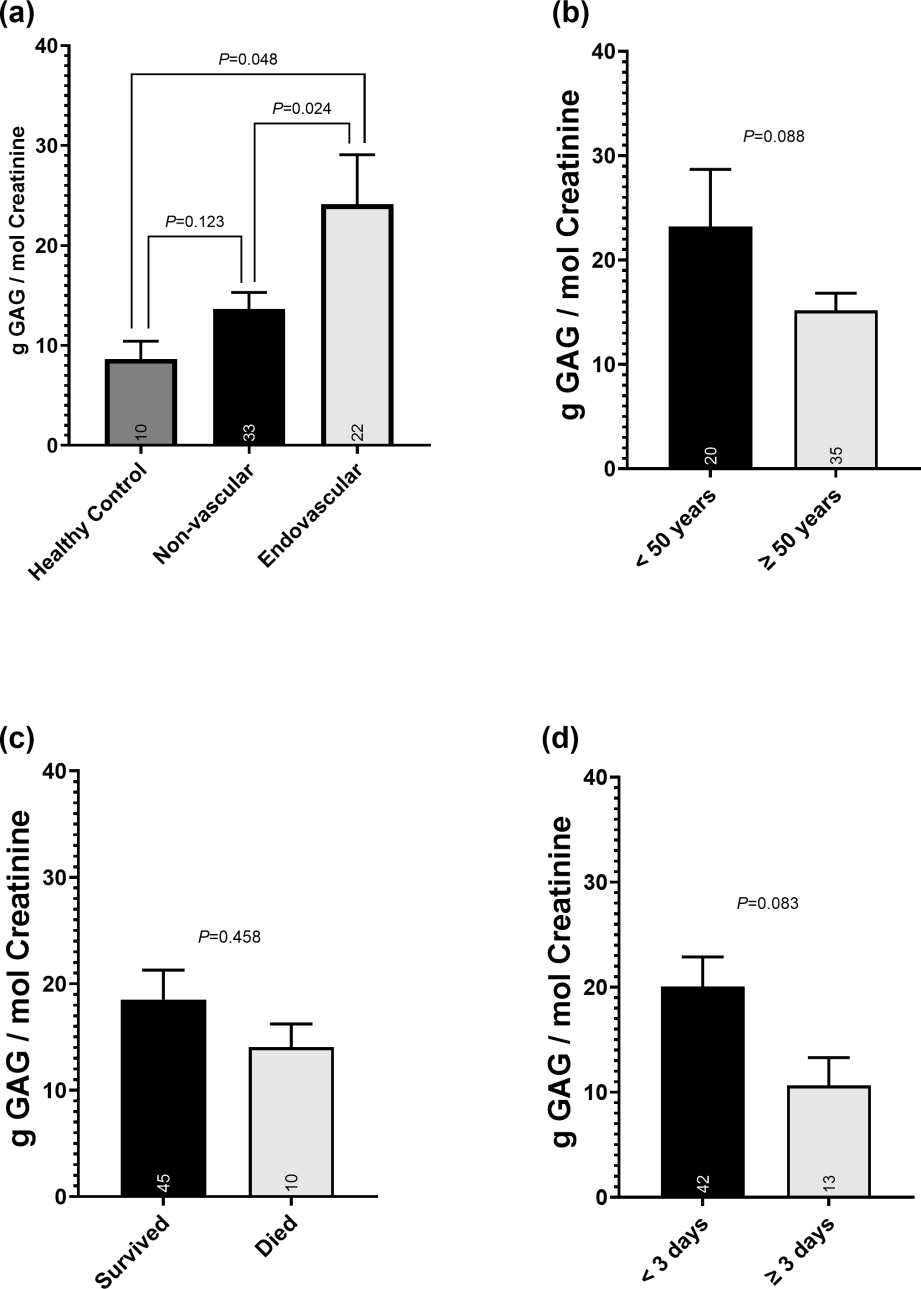
Urine GAG content from patients with *S. aureus* bacteraemia analysed by (a) healthy control and non-vascular and endovascular source, (**b**) age <50 vs ≥50 years, (**c**) survival vs death at 30 days and (d) duration of bacteraemia <3 vs ≥3 days. Data were compared by Student’s t-test.

Patients with catheter-associated bacteraemia and urinary source bacteraemia (e.g. pyelonephritis) were excluded. Table 1 presents patient demographics by cohorts of endovascular and non-vascular bacteraemia source. Endovascular bacteraemic patients were younger with less comorbid conditions by the Charlson index (*P*<0.001). Patients with non-vascular sources were more likely to develop anaemia with bacteraemia during hospitalization (52% vs 23%; *P*=0.049). Shown in Fig. 1(a), endovascular infections (*n*=22, 24.11±4.9 g GAG/mol creatinine) had increased GAG content in the urine compared to non-vascular infections (*n*=33, 13.65±1.6 g GAG/mol creatinine, *P*=0.024) and healthy controls (*n*=10, 8.63±5.67 g GAG/mol creatinine, *P*=0.048). GAG content in younger patients (<50 years of age) versus older patients (≥50 years) was not significantly different (Fig. 1b; *P*=0.0816). GAG was similar between patients who survived versus died at 30 days (*P*=0.458) and in cases of persistent versus resolving bacteraemia (*P*=0.826; Fig. 1c, d). There was no difference in GAG content between male versus female patients (data not shown)

**Table 1.** Comparison of patient characteristics stratified according to endovascular source

Characteristic	Endovascular (*n*=22)	Non-vascular (*n*=33)	*P* value*
Demographics			
Age (years), median (IQR)	34.5 (29.75–63)	62 (56–69)	**<0.001**
Male	13 (59)	20 (61)	>0.99
Comorbidity			
Charlson comorbidity index, median (IQR)	0.5 (0–2.75)	5 (3–8)	**<0.001**
Diabetes	1 (5)	17 (52)	**<0.001**
Moderate/severe CKD	2 (9)	7 (21)	0.29
ICU admission	3 (14)	5 (15)	>0.99
Pitt bacteraemia score, median (IQR)	1 (0.25–2)	1 (0–2)	0.57
WBC count, mean (IQR)†	15.2 (10.5–18.3)	16.6 (8.2–23.6)	0.58
RBC (IQR)	3.81 (3.2–4.5)	3.72 (3.2–4.1)	0.68
Hgb (IQR)	11.15 (9.6–12.6)	11.14 (9.6–13)	0.98
HCT (IQR)	33.5 (29–37)	33.1 (28.8–36.3)	0.83
PLT (IQR)	185.9 (90–256.8)	180.9 (88–230)	0.87
Anaemia during hospital stay	5 (23)	17 (52)	**0.049**
Blood culture time-to-positivity (h), mean (IQR)	16.1 (9.9–16.9)	14.1 (10.7–15)	0.43
Persistent bacteraemia‡	5 (23)	5 (15)	0.50
Mortality			
30 days	5 (23)	5 (15)	0.50
1 year	5 (23)	13 (39)	0.25

Data are presented as *n* (%) unless otherwise indicated. Significant *P* values are bolded.

*Fisher’s exact test or Student’s t-test.

†Initial total white blood cell count at presentation/time of first positive blood culture (10,000 cells/cm3).

‡Blood cultures remaining positive for growth ≥3 days after initial blood culture were classed as persistent.

CKD, Chronic Kidney Disease; HCT, haematocrit; Hgb, haemoglobin; ICU, Intensive Care Unit; IQR, Interquartile Range; PLT, Platelets; RBC, Red Blood Cell; WBC, White Blood Cell.

**Fig. 2.**
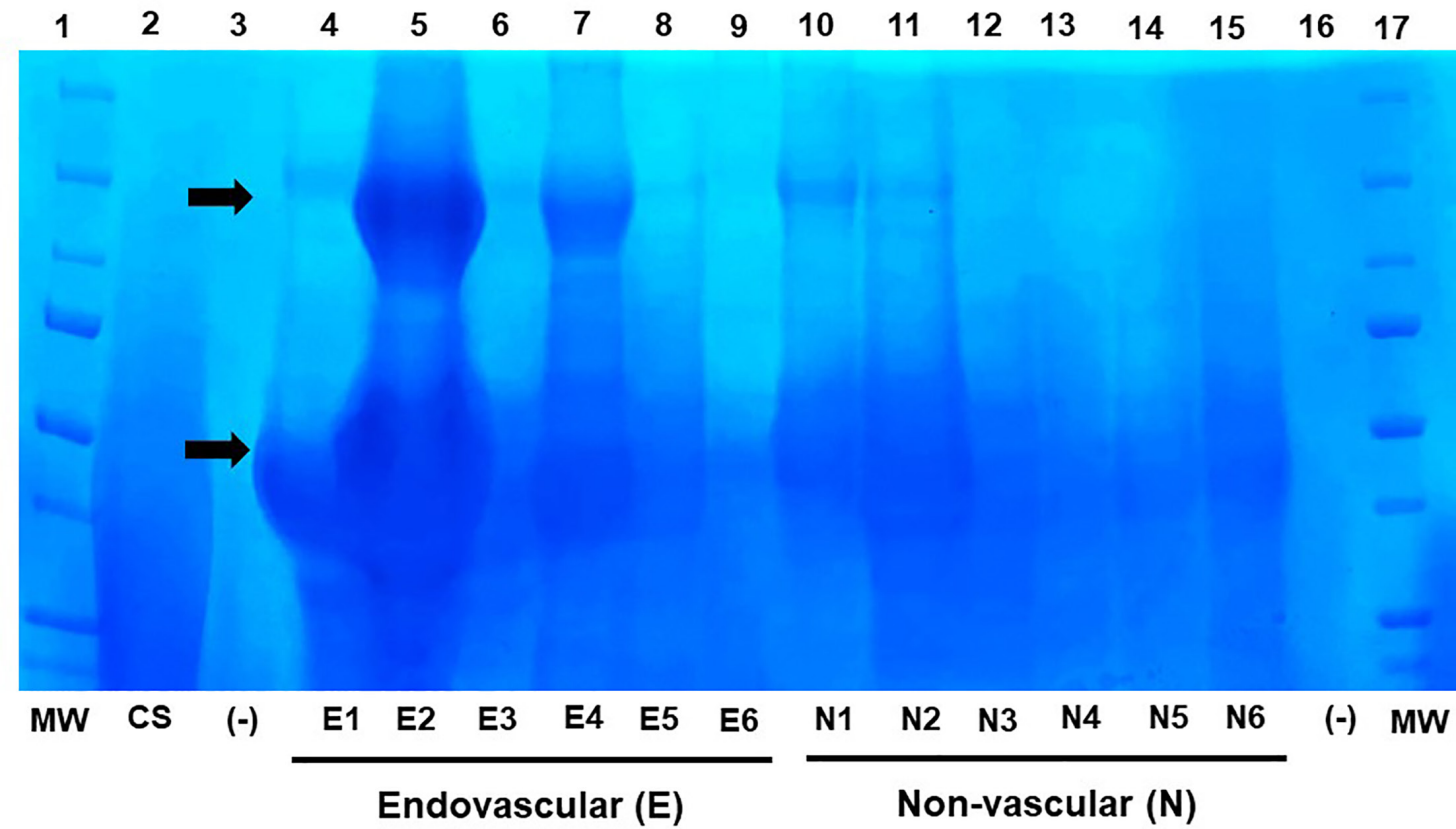
Agarose gel electrophoresis of urinary GAGs from six patients each with *S. aureus* bacteraemia from endovascular (E) and non-vascular (N) sources. The GAGs were extracted from urine as described in Methods and stained by Alcian Blue 8GX. Lanes 1 and 17, molecular weight (MW) ladder; lane 2, chondroitin sulphate (CS); lanes 3 and 16, negative control of buffer and loading dye (-); lanes 4–9, endovascular source patients; lanes 10–15, non-vascular source patients. The arrows indicate areas of high presence of separated GAGs in the endovascular group.

In the randomly selected urine samples from endovascular and non-vascular source patients (*n*=6 per group) for GAG visualization, the total amounts of GAGs appear higher in the endovascular group, consistent with the overall DMMB results, and a differential presence of the varying sulphated glycans was noted (Fig. 2)

## Discussion

This first of its kind pilot study suggests that increased endothelial cell damage increases urine GAG content in cases of *S. aureus* bacteraemia where the primary source is endovascular. These findings are consistent with results from a clinical trial showing increased indicators of glycocalyx shedding (including syndecan-1, hyaluronan and heparan sulphate) in the plasma following cardiac valve surgery [19]. Furthermore, the glycocalyx has intrinsic anti-adherence properties to protect against bacterial infection and implantation, which likely serves a role preventing bacterial attachment/invasion as well as the innate host immune response [20]. Based on the link we observed between endovascular source and creatinine-adjusted high concentrations of GAG in the urine, it appears likely that *S. aureus* is responsible for the destruction and shedding of the glycocalyx. In assessing urinary GAG shedding for other patients and infection metrics, we did not observe any significant associations and therefore cannot currently correlate GAG levels with patient cohorts of age, infection duration or mortality.

Of interest, however, is that GAG content on average was higher in younger populations (<50 years old) despite a lack of statistical significance. It is worth noting that right-sided endocarditis is highly associated with injection-drug use, which in turn is a more common risk behaviour in younger adults. We hypothesize that younger patients can withstand and therefore present to the hospital with higher bacterial burdens in the blood. Older patients with weaker innate immune responses would not only allow the establishment of an infection with a lower bacterial burden but would also not be able to compensate for established higher infection burdens prior to succumbing to illness. Further complicating this analysis is the higher level of comorbidities in our non-vascular infection group, which may increase their baseline GAG shedding in the urine and lead to an under-estimation of the effect size of the endovascular source of infection on GAG presence. The association of urine GAG levels and age in *S. aureus* bacteraemia is an area of future interest and investigation.

The diagnosis of the *S. aureus* bacteraemia source can be difficult. This includes the challenges involved in obtaining transthoracic/transoesophageal echocardiograms for definitive endocarditis (i.e. poor imaging, patient sedation risks and passage of time in scheduling and performing the tests). Furthermore, it may take several days of bacteraemia before definitive echocardiographic findings of endocarditis develop, with repeat testing recommended in high-risk cases [21]. With the high mortality rate of endovascular sources, including endocarditis, an earlier, non-invasive indicator to identify such cases is desperately needed. The rapid detection and assessment of GAG content in the urine, combined with the low cost of reagents and technologies required, suggest that it could be an affordable diagnostic indicator for early deployment of more potent antimicrobial therapies.

The GAGs commonly identified as the major constituents excreted in the urine due to glycocalyx degradation include heparan sulphate, chondroitin sulphate, keratan sulphate and dermatan sulphate [20]. These sulphated GAGs confer function, where the composition of the endothelial glycocalyx plays an important role throughout the body’s vasculature, from maintaining the stability and integrity of the blood-brain barrier to influencing cancer progression and growth [22,23]. While the overall concentration of urine GAGs can vary widely depending on disease state, the composition of shed GAGs has also been shown to vary. In healthy controls, urine from younger patients (ages ≤19 years old) predominantly contained chondroitin sulphate, but as age increased, the proportion of chondroitin sulphate decreased with a coinciding increase in heparan sulphate [3]. Shed GAG proportions have also been shown to change due to disease severity. For instance, as the severity of illness due to malaria increased, there was a change in urine GAG composition such that heparan sulphate and chondroitin sulphate increased and dermatan sulphate concentration decreased [3]. Despite these differences in GAG composition, overall GAG in the urine, measured by the DMMB assay and adjusted for creatinine, was significantly associated with parasite biomass and severity. Of note, we visualized different GAG components in urine in endovascular source *S. aureus* bacteraemia in our select sample of patients. Differentiating and precisely quantifying these GAG components is feasible through MS analysis, and we are interested to explore these individual GAGs in the urine of patients with *S. aureus* bacteraemia to further refine this signature to specific shed glycocalyx components.

The limitations of our study first and foremost include the fact that this was a proof-of-concept pilot study, exploring the measurement of urinary GAG as a potential future diagnostic for stratification of patients with *S. aureus* bacteraemia. It was not intended to establish cut-off values and other details that will be the purpose of larger follow-up studies. However, the mean, median and range of GAGs in ten healthy individuals in this study (8.63, 8.05 and 0.0–19.4 g GAG/mol creatinine, respectively) are useful to identify a baseline range in non-bacteraemic individuals. Furthermore, the study lacked analysis of specific GAG components, which may be possible in future studies by ultra-HPLC-MS/MS [24]. However, isolation of GAGs and separation by gel electrophoresis indicates differences in the overall mass and components of GAGs in endovascular vs non-vascular patients. This study also lacked discrimination between GAG that originated in the bloodstream versus the urine. While we excluded cases of pyelonephritis to minimize this confounder and the fact that urinary source is a lower risk *S. aureus* bacteraemia category not necessarily requiring early detection, filtration of *S. aureus* in the urine in cases of endocarditis is established. It is conceivable that this may release urothelial GAG. However, it stands to reason that such a situation would also serve to add to the sensitivity of total urine GAG (vascular+urothelial) in high-inoculum endovascular bacteraemia. Although our study did not find associations between GAGs and clinical outcomes, this study was not powered to assess these relatively infrequent outcomes, specifically mortality. This study was an exploratory first step, namely, to first identify a link between endocarditis and urinary GAG content. Further analysis in animal studies may solidify this effect. This study also served to lay the justification for a larger study to examine the impact on outcomes. The link between endovascular infections and GAG shedding needs to be explored further, including characterization of the excreted glycocalyx components to further improve this marker in *S. aureus* endovascular infections.

## Abbreviations

DMMB: 1,9-dimethyl-methylene blue
GAG: glycosaminoglycan
IE: infective endocarditis
